# Insights from pharmacovigilance and pharmacodynamics on cardiovascular safety signals of NSAIDs

**DOI:** 10.3389/fphar.2024.1455212

**Published:** 2024-09-04

**Authors:** Shuang Liang, Xianying Wang, Xiuqing Zhu

**Affiliations:** ^1^ Department of Pharmacy, Hebei Medical University Third Hospital, Shijiazhuang, China; ^2^ Key Laboratory of Neurogenetics and Channelopathies of Guangdong Province and the Ministry of Education of China, Guangzhou Medical University, Guangzhou, China; ^3^ Department of Pharmacy, The Affiliated Brain Hospital, Guangzhou Medical University, Guangzhou, China

**Keywords:** NSAIDs, cardiovascular safety signals, FAERS, OpenVigil 2.1, pharmacovigilance, pharmacodynamics, cyclooxygenase-1, cyclooxygenase-2

## Abstract

**Background and Aim:**

Non-steroidal anti-inflammatory drugs (NSAIDs) are commonly used to treat fever, pain, and inflammation. Concerns regarding their cardiovascular safety have been raised. However, the underlying mechanism behind these events remains unknown. We aim to investigate the cardiovascular safety signals and receptor mechanisms of NSAIDs, employing a comprehensive approach that integrates pharmacovigilance and pharmacodynamics.

**Methods:**

This study utilized a pharmacovigilance-pharmacodynamic approach to evaluate the cardiovascular safety of NSAIDs and explore potential receptor mechanisms involved. Data were analyzed using the OpenVigil 2.1 web application, which grants access to the FDA Adverse Event Reporting System (FAERS) database, in conjunction with the BindingDB database, which provides target information on the pharmacodynamic properties of NSAIDs. Disproportionality analysis employing the Empirical Bayes Geometric Mean (EBGM) and Reporting Odds Ratio (ROR) methods was conducted to identify signals for reporting cardiovascular-related adverse drug events (ADEs) associated with 13 NSAIDs. This analysis encompassed three System Organ Classes (SOCs) associated with the cardiovascular system: blood and lymphatic system disorders, cardiac disorders, and vascular disorders. The primary targets were identified through the receptor-NSAID interaction network. Ordinary least squares (OLS) regression models explored the relationship between pharmacovigilance signals and receptor occupancy rate.

**Results:**

A total of 201,231 reports of cardiovascular-related ADEs were identified among the 13 NSAIDs. Dizziness, anemia, and hypertension were the most frequently reported Preferred Terms (PTs). Overall, nimesulide and parecoxib exhibited the strongest signal strengths of ADEs at SOC levels related to the cardiovascular system. On the other hand, our data presented naproxen and diclofenac as drugs of comparatively low signal strength. Cyclooxygenase-1 (COX-1) and cyclooxygenase-2 (COX-2) were identified as central targets. OLS regression analysis revealed that the normalized occupancy rate for either COX-1 or COX-2 was significantly inversely correlated with the log-transformed signal measures for blood and lymphatic system disorders and vascular disorders, and positively correlated with cardiac disorders and vascular disorders, respectively. This suggests that higher COX-2 receptor occupancy is associated with an increased cardiovascular risk from NSAIDs.

**Conclusion:**

Cardiovascular safety of NSAIDs may depend on pharmacodynamic properties, specifically, the percentage of the occupied cyclooxygenase isoenzymes. More studies are needed to explore these relations and improve the prescription process.

## 1 Introduction

Non-steroidal anti-inflammatory drugs, also known as NSAIDs, are commonly used to treat conditions involving fever, pain, and inflammation (Wongrakpanich et al., 2018; Bindu et al., 2020). Given their regular prescriptions for many age groups, especially adults and the elderly, and specifically for chronic conditions, thoroughly evaluating their safety is important (Derry et al., 2016; Varrassi et al., 2020). Although NSAIDs are commonly prescribed in the short run, long-term administration isn’t unusual when dealing with chronic inflammatory diseases (Marcum and Hanlon, 2010; Wehling, 2014). However, concerns have arisen regarding potential risks to cardiovascular events from NSAIDs. This incorporates an increased likelihood of cardiovascular diseases like heart failure, myocardial infarction, and stroke, as well as hypertension (Lindhardsen et al., 2014).

The US Food and Drug Administration (FDA) has emphasized the increased cardiovascular risks associated with the use of NSAIDs, necessitating continuous monitoring. In 2005, the FDA issued an advisory regarding a possible increase in cardiovascular diseases with certain NSAIDs (Castelli et al., 2017). The update further emphasized this notice, highlighting the importance of carefully prescribing NSAIDs, especially long-term in high-risk groups (Tannenbaum et al., 1996; Domper Arnal et al., 2022). Despite these cautions, NSAIDs remain widely used, underscoring the importance of continuing safety tracking (Abou Zeid et al., 2019). NSAIDs exert their effects primarily by inhibiting cyclooxygenase (COX) enzymes, that is, the COX-1 and COX-2. COX-1 is constantly produced and is involved in basic cell functions such as gastric protection and platelet aggregation. COX-2, on the other hand, is inducible and essential for inflammation and pain perception. The inhibition of these enzymes leads to both the therapeutic benefits and adverse effects of NSAIDs. Non-selective NSAIDs act on both the COX-1 and the COX-2 enzymes thus producing undesirable effects on the gastrointestinal as well as the cardiovascular systems; on the other hand, there is a probability that with the use of selective COX-2 inhibitors gastrointestinal effects might be avoided but at the price of having somewhat more cardiovascular manifestations (Grosser et al., 2006).

Substantial monitoring of cardiovascular risks linked with NSAIDs remains necessary. Several past studies have linked NSAID use to an increased risk of cardiovascular diseases (García Rodríguez et al., 2011; Martín Arias et al., 2019; Schjerning et al., 2020). García Rodríguez et al. (2011) have reviewed both randomized controlled trials (RCTs) and observational studies. They found NSAIDs may raise the chances of having a non-fatal heart attack. One large study combined data from multiple observational studies. It reported higher risks of myocardial infarction, abnormal heart rhythms, and heart failure with NSAID use (Schjerning et al., 2020). Another meta-analysis specifically looked at two selective COX-2 inhibitors, celecoxib and etoricoxib. It connected them to an increased risk of cardiovascular diseases (Martín Arias et al., 2019). However, most past research grouped all NSAIDs without analyzing individual drugs or dosage levels (Wu et al., 2018; Ahmed et al., 2019). Studies also mostly focused on just a few types of cardiovascular outcomes. Compared to our research, past studies generally had smaller sample sizes from real-world settings (Kohli et al., 2014; Lin et al., 2017). Some evidence links certain NSAID effects, like how their COX selectivity impacts blood pressure and kidney function, to cardiovascular risks (Harris, 2006; Minhas et al., 2023). However, little data exists on the cardiovascular safety of individual NSAIDs, especially related to these pharmacodynamic properties (Pepine and Gurbel, 2017). Our study included relevant pharmacodynamic data and thus filled this gap in the literature.

**FIGURE 1:**
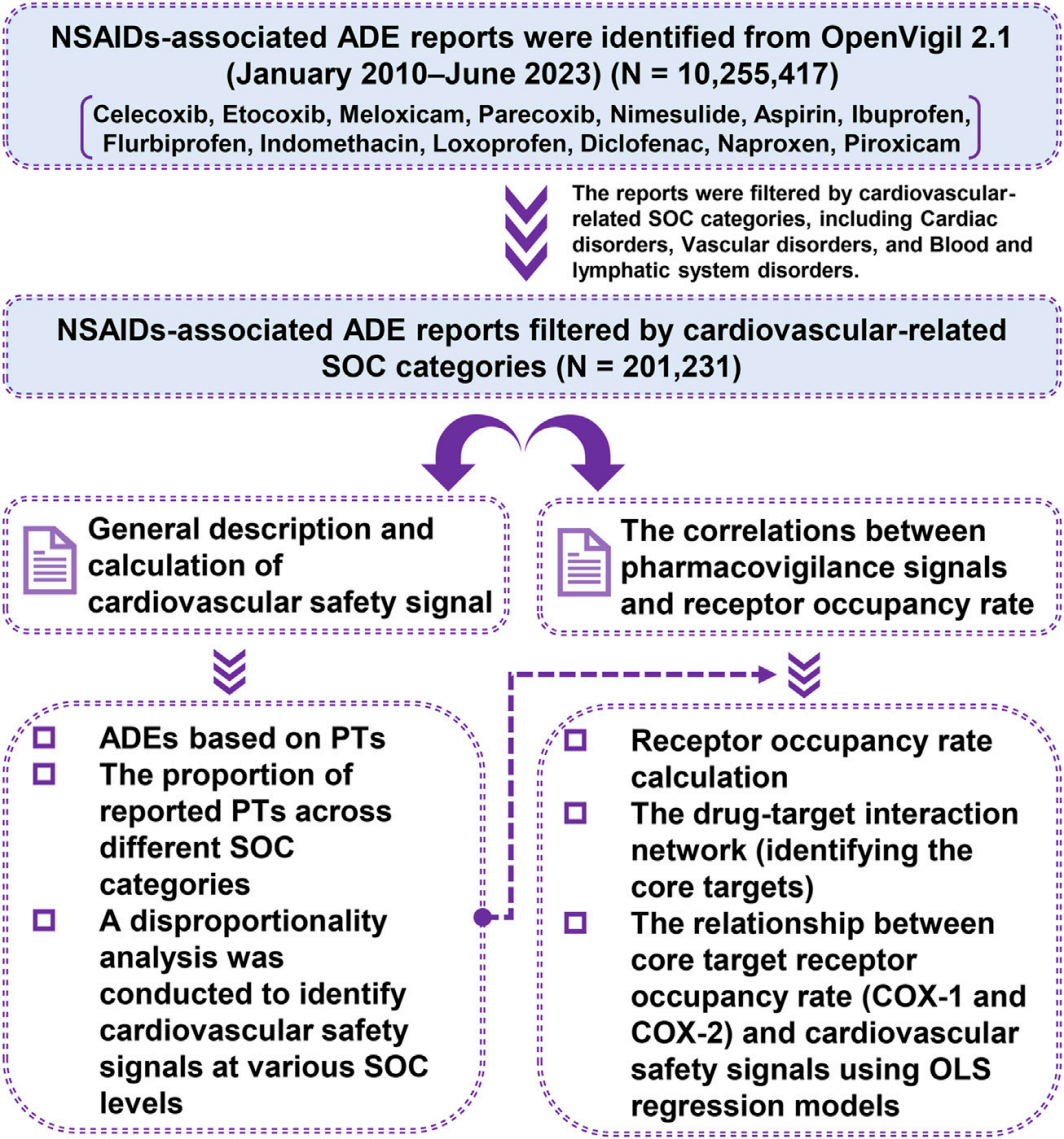
Flow diagram illustrating the sequence of studies conducted.

The goal of our study was to improve understanding of each NSAID’s cardiovascular safety by employing a pharmacovigilance-pharmacodynamic approach. We analyzed the pharmacovigilance data on cardiovascular systems from the FDA Adverse Event Reporting System (FAERS). We also collected the pharmacodynamic data on the receptors of NSAIDs from the BindingDB database. Our objectives were: 1) To see if certain NSAIDs may be linked to safety signals for cardiovascular adverse events; 2) To explore the relationship between reported cardiovascular risk and receptor occupancy rate of NSAIDs. These findings could help clinicians make more informed choices about NSAID use in patient care. Our methods are outlined in Figure 1.

## 2 Materials and methods

### 2.1 Data source

The study used the FDA’s public database of FAERS (https://fis.fda.gov/extensions/FPD-QDE-FAERS/FPD-QDE-FAERS.html) to gather information on suspected adverse drug events (ADEs) related to cardiovascular diseases from use of NSAIDs. This database, which primarily includes reports from the United States but also globally, was accessed through OpenVigil 2.1 (http://h2876314.stratoserver.net:8080/OV2/search/). This platform operated and obtained the cleaned FAERS data, which included verified and normalized drug names. Additionally, it incorporated standard medical terminology for adverse events (AEs) (Sakaeda et al., 2013). The analysis included reports submitted from January 2010 through June 2023. The study focused on clinically used NSAIDs. An NSAID was included in the analysis if it had at least 100 unique reports in the database and at least one report related to cardiovascular events. Ultimately, 13 NSAIDs (aspirin, celecoxib, diclofenac, etoricoxib, flurbiprofen, ibuprofen, indomethacin, loxoprofen, meloxicam, naproxen, nimesulide, parecoxib, and piroxicam) were included in the study and were listed in Table 1. The human receptor information for these NSAIDs, including the inhibitory constants (K_i_), was obtained from the BindingDB database (https://www.bindingdb.org/rwd/bind/index.jsp).

**TABLE 1:** Non-steroidal anti-inflammatory drugs (NSAIDs) and their classifications.

Drug name	Structure	Categorization	Cyclooxygenase (COX) targets of inhibition
Aspirin	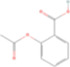	Non-selective COX inhibitors	COX-1, COX-2
Ibuprofen	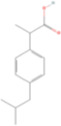	Non-selective COX inhibitors	COX-1, COX-2
Flurbiprofen	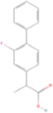	Non-selective COX inhibitors	COX-1, COX-2
Indomethacin	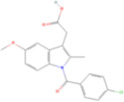	Non-selective COX inhibitors	COX-1, COX-2
Loxoprofen	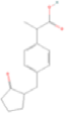	Non-selective COX inhibitors	COX-1, COX-2
Diclofenac	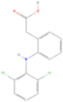	Non-selective COX inhibitors	COX-1, COX-2
Naproxen	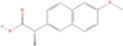	Non-selective COX inhibitors	COX-1, COX-2
Piroxicam	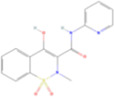	Non-selective COX inhibitors	COX-1, COX-2
Celecoxib	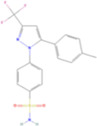	Selective COX-2 inhibitors	COX-2
Parecoxib	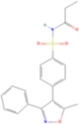	Selective COX-2 inhibitors	COX-2
Meloxicam	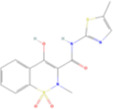	Selective COX-2 inhibitors	COX-2
Etoricoxib	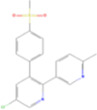	Selective COX-2 inhibitors	COX-2
Nimesulide	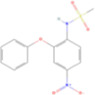	Selective COX-2 inhibitors	COX-2

### 2.2 Detection of cardiovascular risk-related signals

The pharmacovigilance data underwent a process of summarization and sorting based on the number of reported cases for Preferred Terms (PTs) and their corresponding System Organ Classes (SOCs) related to cardiovascular diseases. Three levels of SOCs were identified: heart disease, vascular disease, and blood and lymphatic system diseases. Adverse cardiovascular events were reported for all 13 drugs across all three SOCs, except for parecoxib, which had no reported cases in the category of blood and lymphatic system disorders.

To conduct the disproportionality analysis and explore the association between NSAID use and cardiovascular events, we utilized the ratio imbalance method using a four-square table (refer to Table 2). We employed two algorithms for disproportionate measurement: the Reporting Odds Ratio (ROR) algorithm and the Empirical Bayes Geometric Mean (EBGM) algorithm (Yang et al., 2023). ROR is a simple and highly sensitive method but lacks specificity and is not suitable when the number of target adverse event reports is less than 3 (Jiao et al., 2024). While the method known as EBGM considers the number of reports. This helps provide more stable estimates, especially when report numbers are low (Hunt et al., 2014). Therefore, we combined this method with another one called ROR to create a fuller picture. The calculation formula and the criteria for positive safety signal detection are presented in Table 3.

**TABLE 2:** Disproportionality analysis in pharmacovigilance using a 2 × 2 contingency table.

Drug name	Number of target adverse event reports	Number of other adverse event reports	Total
Target drug	a	b	a + b
Other drugs	c	d	c + d
Total	a + c	b + d	a + b + c + d

Note: “a” represents the number of reports containing both the target drug and target adverse events (AEs). “b” represents the number of reports containing other AEs of the target drug. “c” represents the number of reports containing the target AEs of other drugs. “d” represents the number of reports containing other drugs and other AEs.

**TABLE 3:** Calculation formulas and criteria for positive safety signal detection for the EBGM and ROR algorithms.

Algorithm	Formula	Threshold
EBGM	$EBGM=\frac{\left(aN\right)}{\left[\left(a+b\right)\left(a+c\right)\right]}$ $SE=\sqrt{\frac{1}{a}+\frac{1}{b}+\frac{1}{c}+\frac{1}{d}}$ $95\%\;CI=e^{\ln\left(EBGM\right)\pm 1.96SE}$	the lower bound of 95% CI (EBGM05) > 2
ROR	$ROR=\frac{ad}{bc}$ $SE=\sqrt{\frac{1}{a}+\frac{1}{b}+\frac{1}{c}+\frac{1}{d}}$ $95\%\;CI=e^{\ln\left(ROR\right)\pm 1.96SE}$	a ≥ 3 and the lower bound of 95% CI (ROR05) > 1

Note: EBGM and ROR refer to the Empirical Bayes Geometric Mean algorithm and the Reporting Odds Ratio algorithm, respectively. “a” represents the number of reports containing both the target drug and target adverse events (AEs). “b” represents the number of reports containing other AEs of the target drug. “c” represents the number of reports containing the target AEs of other drugs. “d” represents the number of reports containing other drugs and other AEs. “N” represents the total number of reports, calculated as a + b + c + d. “SE” represents the standard error. “95% CI” represents the 95% confidence interval. “EBGM05” and “ROR05” denote the lower limit of the 95% confidence interval of EBGM for the EBGM algorithm and the lower limit of the 95% confidence interval of ROR for the ROR algorithm, respectively.

### 2.3 Correlations between cardiovascular safety signals and receptor occupancy rate

To quantify the receptor-mediated mechanisms, we selected the receptor occupancy rate, which was computed using the pharmacological receptor theory: Occupancy (%) = 100 × C_U_/(K_i_ + C_U_). The variable C_U_ (nM) refers to the concentration of the unbound drug in the blood; meanwhile, K_i_ (nM) stands for the inhibitory constant of the drug under consideration (Kenakin, 2004; Montastruc et al., 2015; Cepaityte et al., 2021). In the context of the receptor-NSAID interaction analysis presented in the next section, 11 NSAIDs (aspirin, celecoxib, diclofenac, etoricoxib, flurbiprofen, ibuprofen, indomethacin, meloxicam, naproxen, nimesulide, and piroxicam) were applied to the analysis with available K_i_ data for each of them. The C_U_ was estimated using the formula: C_U_ = 1000 × F_U_ × C_T_/MW, where F_U_ depicts the proportion of unbound drug obtained from Drugbank (Harding et al., 2018), C_T_ (ng/mL) signifies the concentration of the drug in the blood, while MW (g/mol) refers to the molecular weight obtained from the drug label (Cepaityte et al., 2021). In this analysis, the total drug concentration in the blood (C_T_) was estimated using the upper limit of the therapeutic reference range of individual NSAIDs, as documented on the drug label (Hiemke et al., 2018). If there were two or more figures available for the target occupancy of the drug to one receptor, the mean receptor occupancy rate was used.

To explore whether cardiovascular safety signals relate to varying receptor occupancy rates, we examined the extent to which there is a connection between the receptor and NSAID network. In this network, the nodes of the network were the receptors and NSAIDs whilst the width of edges in the network represented the average receptor occupancy rate. The target which contained the largest number of degrees in the interaction networks was chosen as a central target and further analyzed. The following OLS regression model was developed to test the hypothesis: the receptor occupancy rate of the core target of NSAIDs had a relationship with the level of cardiovascular pharmacovigilance signal intensity, using normalized receptor occupancy rates and log-transformed signal measures [log (EBGM05) and log (ROR05)] across three cardiovascular SOCs. As long as the *P*-value for either algorithm is less than 0.05, the correlation is considered statistically significant. Last but not least, a scatter plot was utilized to visually illustrate the significant association between the normalized receptor occupancy rate and the log-transformed number of pharmacovigilance signal intensity.

All statistical analyses were performed using Python (3.8.13) and packages, including pandas (1.5.3), numpy (1.23.0), scipy (1.8.1), scikit-learn (1.3.2), statsmodels (0.14.0), network (3.1), matplotlib (3.4.3), seaborn (0.12.2), and proplot (0.9.7).

## 3 Results

### 3.1 Data overview

**FIGURE 2:**
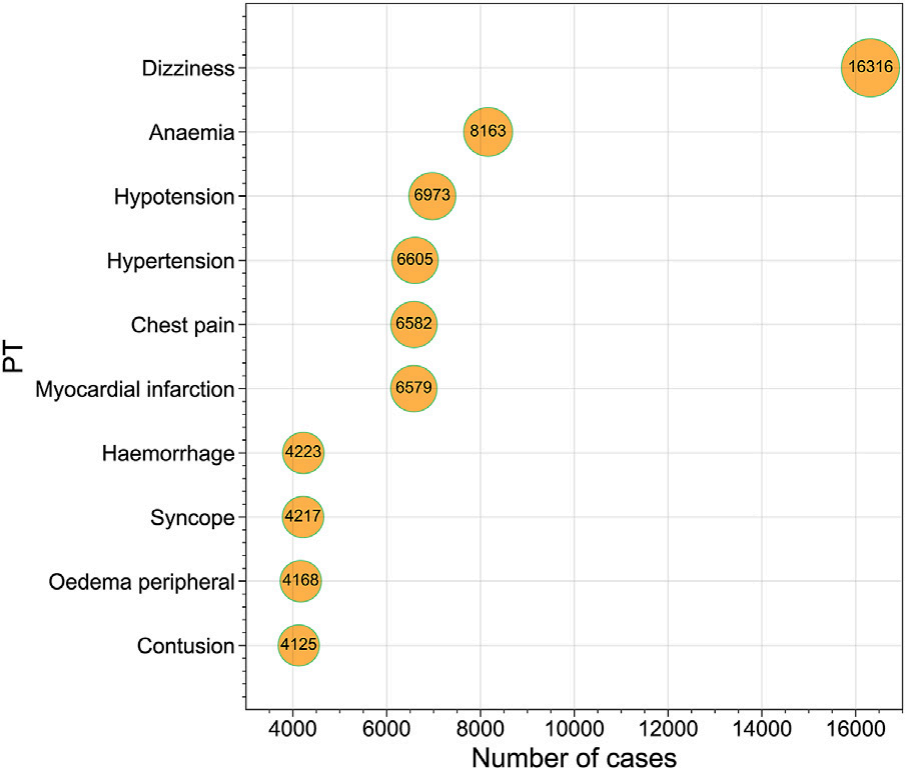
Bubble chart presenting the top 10 Preferred Terms (PTs) ranked by the number of reported cases.

**FIGURE 3:**
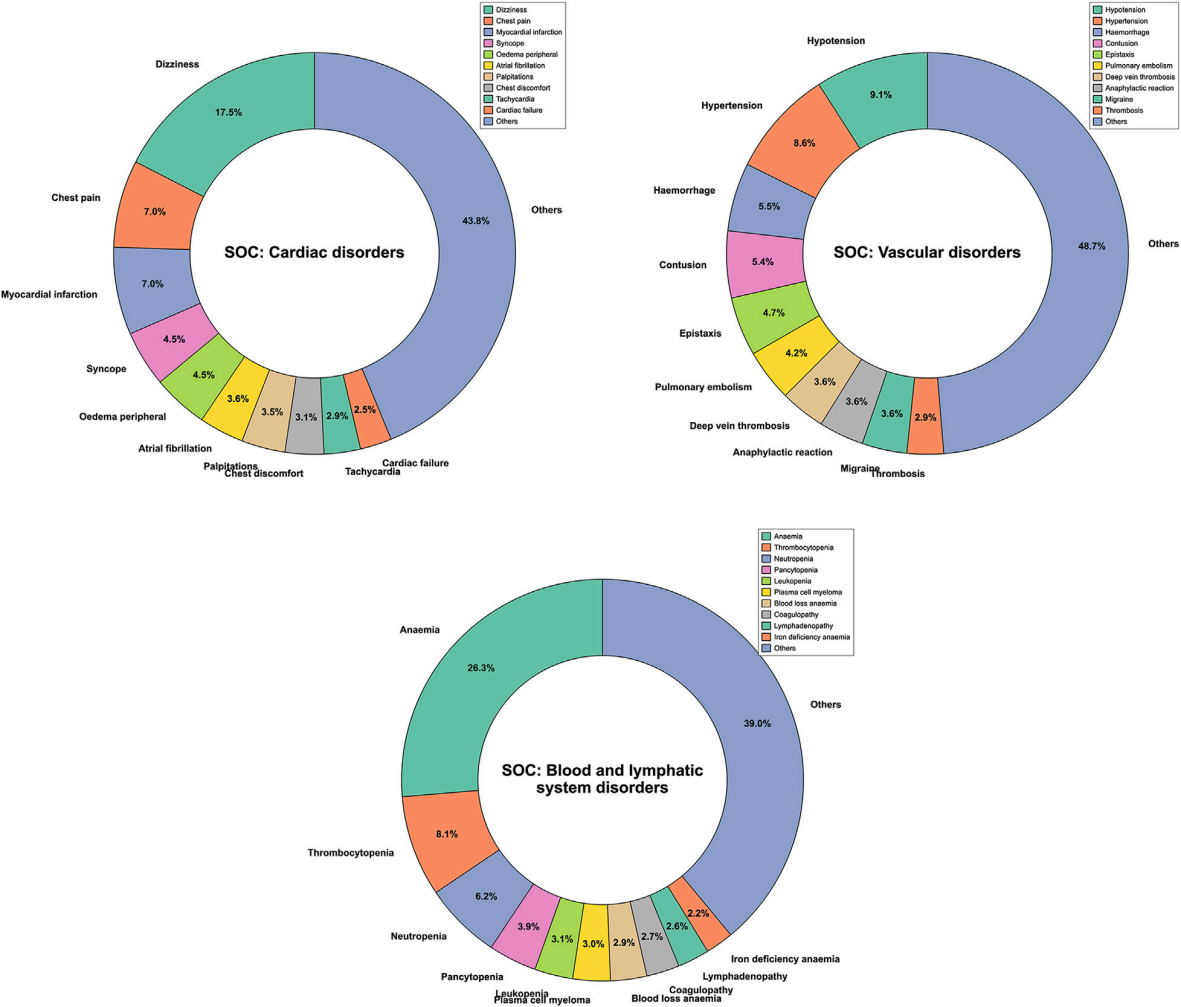
Pie chart displaying the distribution of reported Preferred Terms (PTs) across different System Organ Classes (SOCs).

A total of 10,255,417 ADE reports for 13 NSAIDs were obtained from OpenVigil 2.1 (Please refer to Supplementary Table S1 for more details). These reports encompassed 27 different SOCs and 12,165 PTs. Following filtration based on cardiovascular-related SOC categories, a total of 201,231 ADE reports were ultimately screened (Figure 1). This subset included 93,453 PTs related to cardiac disorders, 76,766 PTs related to vascular disorders, and 31,012 PTs related to blood and lymphatic system disorders. We compiled the reported cases of adverse cardiovascular events across the different NSAIDs and ranked the PTs based on case numbers. Figure 2 displays the top ten PTs associated with cardiovascular events among the 13 NSAIDs. Among these, dizziness was the most frequently reported PT (16,316 cases), followed by anemia (8,163 cases), hypotension (6,973 cases), hypertension (6,605 cases), chest pain (6,582 cases), myocardial infarction (6,579 cases), hemorrhage (4,223 cases), syncope (4,217 cases), oedema peripheral (4,168 cases), and contusion (4,125 cases) (Figure 2). The distribution of reported cases for PTs under the three cardiovascular-related SOCs is illustrated in the pie charts (Figure 3). For cardiac disorders, the top three reported PTs were dizziness (17.5%), chest pain (7.0%), and myocardial infarction (7.0%). In the case of vascular disorders, the leading PTs were hypotension (9.1%), hypertension (8.6%), and hemorrhage (5.5%). Regarding blood and lymphatic system disorders, the most prevalent PTs were anemia (26.3%), thrombocytopenia (8.1%), and neutropenia (6.2%).

### 3.2 Signal detection at SOC level for cardiovascular safety

**FIGURE 4:**
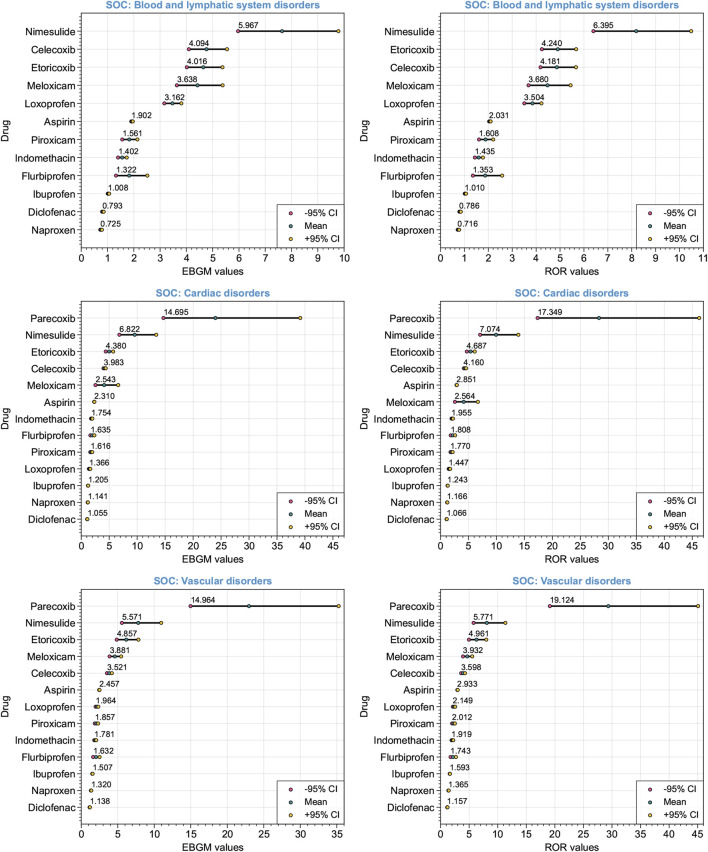
Forest plot depicting the ranking of detected signals at various System Organ Classes (SOCs) levels associated with the cardiovascular system. The Empirical Bayes Geometric Mean (EBGM) algorithm and the Reporting Odds Ratio (ROR) algorithm were utilized. Red and yellow dots represent the −95% and +95% confidence intervals, respectively. The mean EBGM or ROR values are indicated by green dots.

Significant safety signals were identified through signal detection analysis using the EBGM and ROR algorithms across the three cardiovascular-related SOCs. Figure 4 illustrates the ranking of detected signals at different SOC levels using the EBGM and ROR algorithms. Consistent disproportionality signals were identified by integrating the discriminative criteria of positive safety alert signals based on the EBGM and ROR algorithms. Specifically, this included the lower limit of the 95% confidence interval of EBGM above 2 (EBGM05 > 2) for the EBGM algorithm, or the lower limit of the 95% confidence interval of ROR above 1 (ROR05 > 1) for the ROR algorithm, along with the count of reports that include both the target drug and target AEs with a frequency equal to or greater than 3 (*a* ≥ 3) (Table 3). Significant disproportionality signals were found for blood and lymphatic system disorders: nimesulide (*a* = 68, EBGM05 = 5.967, ROR05 = 6.395), celecoxib (*a* = 171, EBGM05 = 4.094, ROR05 = 4.181), etoricoxib (*a* = 194, EBGM05 = 4.016, ROR05 = 4.240), meloxicam (*a* = 102, EBGM05 = 3.638, ROR05 = 3.680), and loxoprofen (*a* = 508, EBGM05 = 3.162, ROR05 = 3.504). Significant disproportionality signals also emerged for six NSAIDs on cardiac disorders: parecoxib (*a* = 19, EBGM05 = 14.695, ROR05 = 17.349), nimesulide (*a* = 35, EBGM05 = 6.822, ROR05 = 7.074), etoricoxib (*a* = 237, EBGM05 = 4.380, ROR05 = 4.687), celecoxib (*a* = 1763, EBGM05 = 3.983, ROR05 = 4.160), meloxicam (*a* = 17, EBGM05 = 2.543, ROR05 = 2.564), and aspirin (*a* = 53,659, EBGM05 = 2.310, ROR05 = 2.851). The aforementioned six NSAIDs also showed consistent disproportionality signals for vascular disorders: parecoxib (*a* = 27, EBGM05 = 14.964, ROR05 = 19.124), nimesulide (*a* = 35, EBGM05 = 5.571, ROR05 = 5.771), etoricoxib (*a* = 69, EBGM05 = 4.857, ROR05 = 4.961), meloxicam (*a* = 133, EBGM05 = 3.881, ROR05 = 3.932), celecoxib (*a* = 520, EBGM05 = 3.521, ROR05 = 3.598), and aspirin (*a* = 42,990, EBGM05 = 2.457, ROR05 = 2.933). Overall, nimesulide and parecoxib exhibited the strongest signal strengths of ADEs at SOC levels related to the cardiovascular system, reaching statistical significance. Conversely, naproxen and diclofenac showed weak signal strengths of ADEs, without any observed disproportionality.

### 3.3 Relationship between core target receptor occupancy rate and cardiovascular safety signals

**FIGURE 5:**
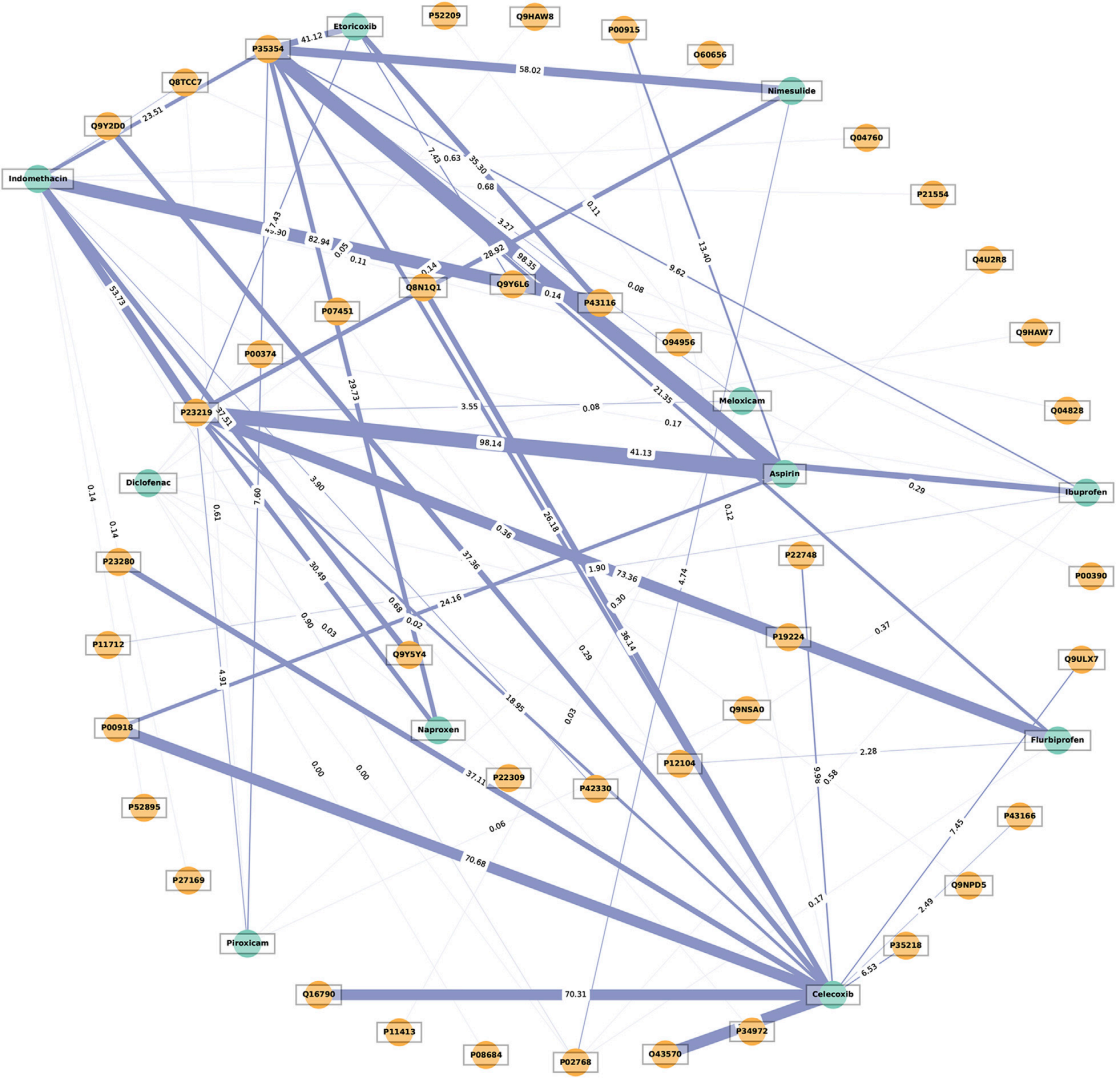
The network illustrates the interaction between receptors and non-steroidal anti-inflammatory drugs (NSAIDs). The thickness of the blue lines represents the average receptor occupancy rate. The green circles represent the NSAIDs, while the orange circles represent the targets.

A network graph was constructed to illustrate the interactions between NSAIDs (green circles) and their targets (orange circles), highlighting the significance of these interactions. The thickness of the connecting blue lines corresponds to the average occupancy rate of the targets (Figure 5). Cyclooxygenase-1 (COX-1, UniProt protein ID: P23219) and cyclooxygenase-2 (COX-2, UniProt protein ID: P35354) have emerged as central targets in the receptor-NSAID interaction network, each with a degree of 10. It is noteworthy that for COX-1, aspirin displayed the highest average occupancy rate at 98.14%, followed by flurbiprofen at 73.36%, and indomethacin at 53.73%. On the other hand, for COX-2, aspirin showed the highest average occupancy rate at 98.35%, followed by nimesulide at 58.02%, and etoricoxib at 41.12%. The UniProt protein ID numbers and their corresponding meanings were provided in Supplementary Table S2.

**FIGURE 6:**
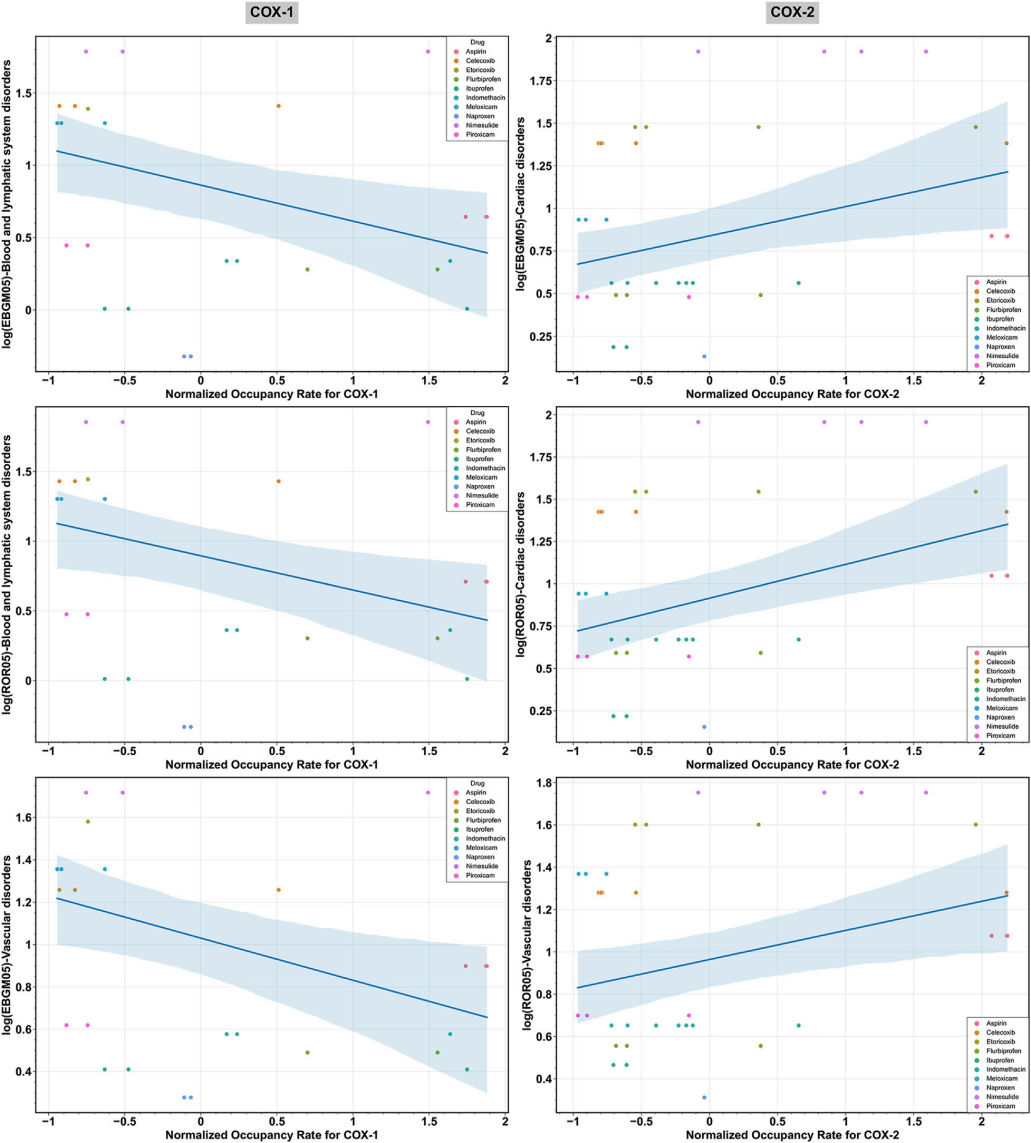
Scatter plot illustrating the significant relationships between the log-transformed signal measures [log (EBGM05) and log (ROR05)] across various System Organ Classes (SOCs) and the normalized receptor occupancy rates of cyclooxygenase-1 (COX-1) and cyclooxygenase-2 (COX-2) for non-steroidal anti-inflammatory drugs (NSAIDs). “EBGM05” and “ROR05” represent the lower limit of the 95% confidence interval for EBGM using the EBGM algorithm and ROR using the ROR algorithm, respectively.

OLS regression models were utilized to examine the correlation between core target receptor occupancy rates and cardiovascular safety signals. Table 4 demonstrates a weak negative correlation between the normalized COX-1 receptor occupancy rate for NSAIDs and the log (EBGM05) and log (ROR05) for blood and lymphatic system disorders, as well as the log (EBGM05) for vascular disorders, with statistical significance. Conversely, a weak positive correlation was observed between the normalized COX-2 receptor occupancy rate for NSAIDs and the log (EBGM05) and log (ROR05) for cardiac disorders, along with the log (ROR05) for vascular disorders, with statistical significance. No significant associations were found between the COX-1 occupancy rate and log-transformed signal measures for cardiac disorders, nor between the COX-2 occupancy rate and log-transformed signal measures for blood and lymphatic system disorders. Figure 6 presents scatter plots illustrating the log (EBGM05) or log (ROR05) for these significantly correlated SOCs among 10 NSAIDs interacting with COX-1 or COX-2 in the receptor-NSAID interaction network, alongside their corresponding normalized COX-1 or COX-2 occupancy rates. NSAIDs such as aspirin, nimesulide, etoricoxib, naproxen, celecoxib, indomethacin, flurbiprofen, ibuprofen, piroxicam, and meloxicam are depicted by colored dots. This suggests that higher COX-2 receptor occupancy is associated with an increased cardiovascular risk from NSAIDs, while higher COX-1 receptor occupancy is linked to a reduced cardiovascular risk from NSAIDs.

**TABLE 4:** Ordinary least squares (OLS) regression analysis: Normalized receptor occupancy rate of cyclooxygenase-1 (COX-1) and cyclooxygenase-2 (COX-2) for non-steroidal anti-inflammatory drugs (NSAIDs) and cardiovascular safety signals [log (EBGM05) and log (ROR05)] across various System Organ Classes (SOCs).

COX	SOC	EBGM algorithm				ROR algorithm			
		Coefficient [2.5% CI, 97.5% CI]	t-value	*P*-value	*R* ^2^ (%)	Coefficient [2.5% CI, 97.5% CI]	t-value	*P*-value	*R* ^2^ (%)
COX-1	Blood and lymphatic system disorders	−0.2492 [–0.475, −0.023]	−2.255	**0.032**	14.5	−0.2454 [–0.478, −0.013]	−2.154	**0.039**	13.4
	Cardiac disorders	−0.1585 [–0.362, 0.045]	−1.594	0.121	7.8	−0.1227 [–0.327, 0.082]	−1.226	0.230	4.8
	Vascular disorders	−0.1991 [–0.371, −0.027]	−2.366	**0.025**	15.7	−0.1671 [–0.337, 0.002]	−2.013	0.053	11.9
COX-2	Blood and lymphatic system disorders	0.1233 [–0.066, 0.312]	1.316	0.195	3.9	0.1378 [–0.055, 0.330]	1.443	0.156	4.6
	Cardiac disorders	0.1716 [0.017, 0.326]	2.242	**0.030**	10.5	0.1996 [0.050, 0.349]	2.699	**0.010**	14.5
	Vascular disorders	0.1159 [–0.025, 0.256]	1.664	0.103	6	0.1377 [0.004, 0.271]	2.078	**0.044**	9.1

Note: EBGM and ROR refer to the Empirical Bayes Geometric Mean and the Reporting Odds Ratio algorithms, respectively. “EBGM05” and “ROR05” denote the lower limit of the 95% confidence interval of EBGM for the EBGM algorithm and the lower limit of the 95% confidence interval of ROR for the ROR algorithm, respectively. Bold *P*-values indicate statistical significance. CI represents confidence interval.

## 4 Discussion

This study aimed to assess the cardiovascular safety of NSAIDs by integrating pharmacovigilance and pharmacodynamics approaches. Using the OpenVigil 2.1 platform, a comprehensive pharmacovigilance analysis was conducted on a substantial number of real-world AE reports obtained from the FAERS database, which provided data on the use of 13 NSAIDs. Additionally, a pharmacodynamics analysis was performed utilizing the BindingDB database, which offered valuable target information on drug-target interactions. The study initially provided an overview of the AEs associated with NSAIDs, including the ordering of PTs related to cardiovascular events and the proportional distribution of cardiovascular-related PTs within different SOCs. Consequently, the disproportionality analysis to search for cardiovascular safety signals at different SOC levels was performed. Therefore, for the current study, the possible pharmacological receptor mechanisms through which cardiovascular safety signals were found were investigated. This entailed determining the receptor occupancy rate and pinpointing the core targets through the drug-target interaction network. Last of all, given the hypotheses that the receptor occupancy rate of the core target, COX-1 and/or COX-2 correlated with cardiovascular safety signaling, an OLS regression model examined this relationship. Thus, combining the findings of pharmacovigilance and pharmacodynamics, this study provides significant information about the cardiovascular safety signals of NSAIDs. In summary, the study adds to the body of knowledge and evaluation of the cardiovascular risks of these commonly used NSAIDs.

Whereas earlier studies have focused on showing a risk for cardiovascular events connected with NSAID exposure determined by meta-analyses of RCTs and observational studies, there is still limited and inconclusive data available regarding individual NSAIDs (Bally et al., 2017; Schjerning et al., 2020). Multiple studies have indicated that naproxen carries the lowest cardiovascular risk among NSAIDs. In contrast, the utilization of COX-2 inhibitors or diclofenac in high-risk patients is linked to a greater occurrence of cardiovascular events (McGettigan and Henry, 2011). However, a meta-analysis revealed that all NSAIDs, including naproxen, are linked to an augmented risk of acute myocardial infarction. Conversely, the risk associated with celecoxib does not seem to surpass that of traditional NSAIDs (Bally et al., 2017). One of the objectives of our investigation was to elucidate the correlation between specific NSAIDs and the augmented risk of cardiovascular events founded upon real-world evidence.

In our analysis, we focused on the top 10 PTs associated with cardiovascular events, and the results revealed that dizziness was the most commonly reported AE. A study conducted on the adverse effects of analgesics, including 11 NSAIDs, among elderly individuals yielded consistent findings with our study, highlighting dizziness as the most frequently reported AE, except for gastrointestinal reactions (McDonald et al., 2018). However, the prevalence of these AEs varied across different categories of cardiovascular-related SOCs. Cardiac disorders were predominantly associated with dizziness as the most reported PT, whereas vascular disorders were primarily linked to hypotension as the leading PT. In the case of blood and lymphatic system disorders, anemia was the most prevalent AE.

Disproportionality analysis revealed significant safety signals associated with the reporting of cardiovascular-related AEs. Among blood and lymphatic system disorders, nimesulide demonstrated the strongest signal, while naproxen exhibited the weakest signal. In both the cardiac disorders and vascular disorders systems, parecoxib manifested the strongest signal, whereas diclofenac evinced the weakest signal. Recent reports on the cardiovascular risk profile of NSAIDs suggest that the risk of adverse cardiovascular events varies among different NSAIDs. Naproxen appears to carry a relatively lower cardiovascular risk compared to other NSAIDs (Minhas et al., 2023). Research conducted in Denmark also indicates that naproxen exhibits a more favorable cardiovascular risk profile (Bech-Drewes et al., 2024). A systematic review and meta-analysis indicated that postoperative administration of parecoxib may elevate the risk of cardiovascular complications (Aldington et al., 2005). Our real-world research findings from the FAERS database align with the aforementioned conclusions.

**FIGURE 7:**
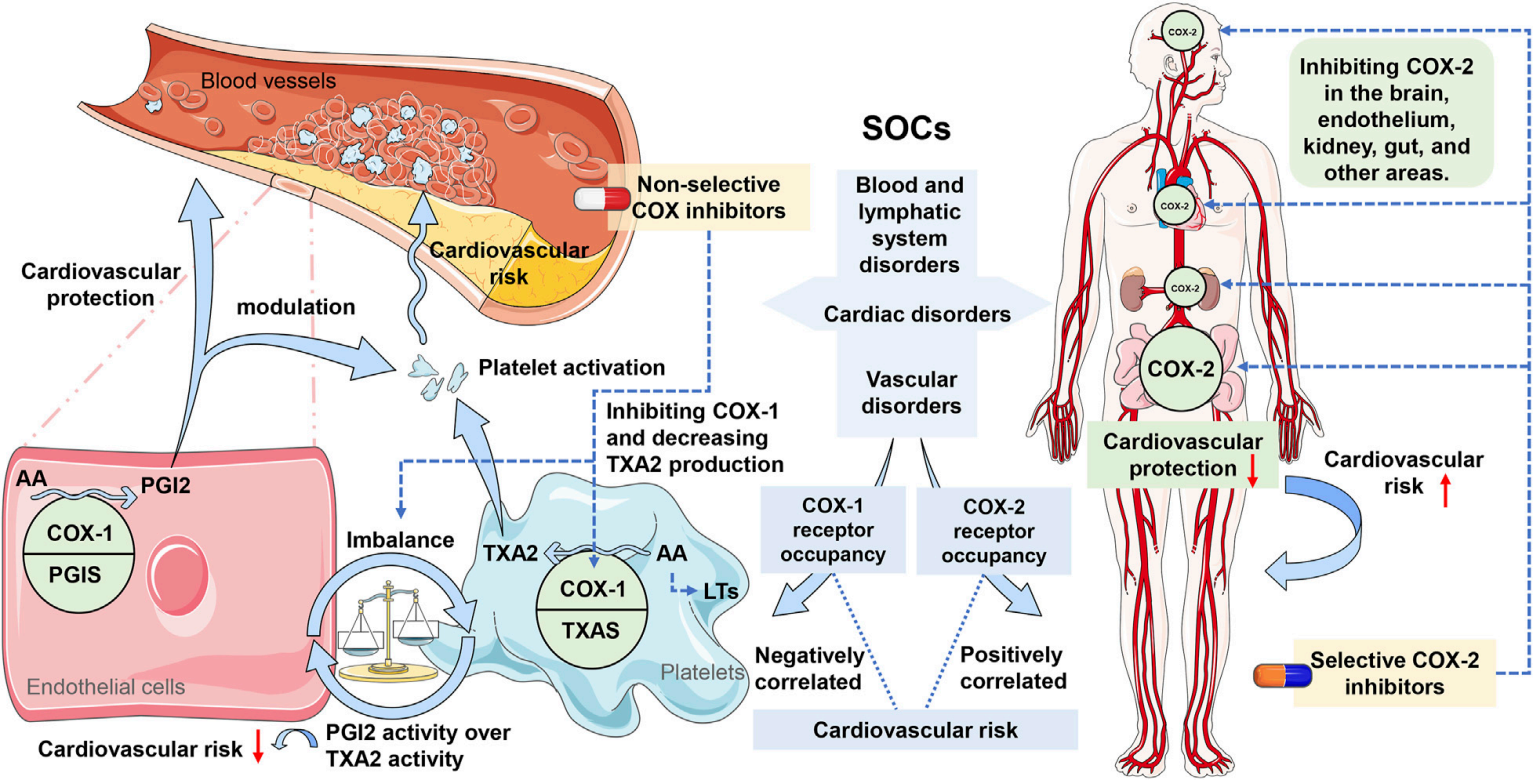
Potential molecular mechanisms of non-steroidal anti-inflammatory drugs (NSAIDs) associated with cardiovascular safety signals. Note: COX, cyclooxygenase; COX-1, cyclooxygenase-1; COX-2, cyclooxygenase-2; SOCs, System Organ Classes; AA, arachidonic acid; PGIS, prostacyclin synthase; TXAS, thromboxane synthase; PGI2, prostacyclin; TXA2, thromboxane A2; LTs, leukotrienes.

Several receptor mechanisms have been proposed to elucidate the occurrence of NSAID-related cardiovascular events. Following the obtained outcomes of the present research, it is possible to assume that the factors that are related to the pharmacodynamics of NSAIDs, particularly their ability to cause COX-1 and COX-2 occupancy, may influence cardiovascular safety levels. A study also gives evidence that affords the hypothesis stating that the degree of specificity of the NSAID to COX-2 in comparison with COX-1 plays a significant role in the cardiovascular hazard (Schjerning et al., 2020). Also, it is shown that the network analysis of the target-NSAID interactions underscores the importance of these interactions. The actual analysis of the estimator obtained by the OLS regression models points towards a statistically significant, albeit relatively weak, negative correlation between the normalized occupancy rate of the COX-1 receptor and the log-transformed signal measures for blood and lymphatic system disorders and vascular disorders, as well as a positive correlation between the normalized occupancy rate of the COX-2 receptor and the log-transformed signal measures for cardiac disorders and vascular disorders. Thus, these divergent cardiovascular risks that are attributed to NSAIDs are believed to be linked to their mechanism of action that inhibits the COX enzymes, which in turn affect the synthesis of prostaglandins (PG) (Funk and FitzGerald, 2007). These enzymes, COX-1 and COX-2, play many important roles in the cardiovascular system, though some of their effects oppose each other (Khan et al., 2019). Any changes that affect the vascular function and platelet aggregation will raise the risk of thrombus formation. As illustrated in Figure 7, COX-1 is found throughout the cardiovascular system, especially in blood vessels and platelets. In healthy blood vessels, COX-1 is mainly located in the endothelial layer where it works with prostacyclin synthase (PGIS) to mostly produce prostacyclin (PGI2). Within platelets, COX-1 combines with thromboxane synthase (TXAS), leading to the primary production of thromboxane A2 (TXA2). This causes platelet aggregation, vasoconstriction, and smooth muscle hypertrophy. Conversely, the COX-1-mediated synthesis of PGI2 in vascular endothelial cells can hinder platelet aggregation, promote vasodilation, inhibit smooth muscle proliferation, and play a significant protective role in the cardiovascular system (Mitchell et al., 2021). Moreover, PGI2 has been found to modulate various prothrombotic stimuli, such as adenosine diphosphate (ADP), epinephrine, collagen, serotonin, thrombin, and TXA2 (Varga et al., 2017; Schjerning et al., 2020). A healthy cardiovascular system typically exhibits higher protective PGI2 activity than TXA2 activity (Mitchell et al., 2021). Consequently, COX-1 inhibitors like aspirin, ibuprofen, and diclofenac suppress TXA2 production more than PGI2 when used. This disrupts the balance between TXA2 and PGI2, resulting in elevated PGI2 activity over TXA2 activity, which offers a degree of protection. It could help to explain the relationship between the increasing COX-1 receptor occupancy with reduced cardiovascular risk from NSAIDs. On the other hand, while COX-2 is expressed in regions of vascular inflammation and disease, it is minimally expressed in most blood vessels and is largely absent from platelets. However, it plays a role in maintaining cardiovascular health in distant areas such as the kidney and endothelium. Evidence suggests that NSAIDs are associated with an increased risk of hypertension and renal impairment that can share their connection with cardiovascular diseases (Schafer, 1995; Braun et al., 2020). The results of the pharmacodynamic analysis indicate that the specific drug-receptor interactions may be some of the factors that cause this. For instance, COX-2 in the kidney safeguards the cardiovascular system by reducing blood pressure and controlling the levels of hypertensive mediators like asymmetric dimethylarginine. Inhibiting renal COX-2 with selective COX-2 inhibitors can elevate blood pressure. Endothelial COX-2 fosters the production of the antithrombotic hormone PGI2, and hindering COX-2 with selective inhibitors raises the risk of cardiac thrombosis (Mitchell et al., 2021). Therefore, this scenario may partially elucidate why selective COX-2 inhibitors show a heightened cardiovascular risk signal, where a greater COX-2 receptor occupancy correlates with an increased likelihood of adverse cardiovascular events, primarily cardiac disorders and vascular disorders.

However, the strength of the correlations mentioned above was found to be weaker for both COX-1 and COX-2, and all NSAIDs have been associated with cardiovascular risks according to systematic reviews and meta-analyses (Bally et al., 2017). Incidental COX-1 blockade by traditional NSAIDs does not reduce the likelihood of cardiovascular side effects (Mitchell et al., 2021). One possible explanation is that non-selective NSAIDs inhibit COX-1 without specificity, while also non-selectively inhibiting COX-2, potentially elevating the risk of cardiovascular events. Another hypothesis is that the inhibition of COX enzymes by NSAIDs could redirect arachidonic acid (AA) to alternative enzymatic pathways, like 5-lipoxygenase (5-LOX) and leukotrienes (LTs), which play roles in atherosclerosis and inflammation processes. NSAIDs’ COX blockade might shift AA towards leukotriene C4 (LTC4) in immune cells and other LTs in endothelial cells, potentially contributing to the observed cardiovascular side effects associated with NSAIDs (Mitchell et al., 2021) (see Figure 7). These weak correlations might also be due to the opinion that cardiovascular events depend on various receptors and mechanisms (Schjerning et al., 2020). For instance, referring to Figure 5, the targets for cardiovascular adverse events tied to etoricoxib and nimesulide involve COX and two other entities, namely albumin (P02768) and prostaglandin E2 (P43116). The approval of the hypotheses of our OLS regression model moreover supports the hypothesis that adverse cardiovascular events caused by NSAIDs are due to the operations of several receptors simultaneously. In addition, it was discovered that drugs with greater selectivity for COX-2 had a higher risk of cardiovascular occurrences, which proves the concept of pharmacodynamic selectivity crucial in assessing drug safety (Bonnesen and Schmidt, 2021; Stiller and Hjemdahl, 2022). In summary, certain drugs may impact cardiovascular function through their interactions with various receptors, particularly those with distinct pharmacodynamic characteristics (Su, 2006; Soslau, 2022). The inhibition of COX-2 by NSAIDs is beneficial for pain relief and inflammation; however, it may disrupt the delicate balance between prothrombotic and antithrombotic factors, thereby increasing the risk of cardiovascular events (Graham, 2006; Rahme and Nedjar, 2007; Marsico et al., 2017; Stiller and Hjemdahl, 2022).

It is vital to notice that both the spontaneous reporting system and the disproportionality analysis are necessary tools in the field of safety monitoring. Our approach of integrating pharmacovigilance data with pharmacodynamic insights aligns with recent strategies employed in studying AEs associated with drugs within the same pharmacological class. It has been useful in explaining the receptor mechanisms underlying AEs reported in safety databases such as in cases of metabolic disturbances or pneumonia by antipsychotics and cardiovascular toxicity of drugs (Reynolds and McGowan, 2017; Cepaityte et al., 2021; Chen et al., 2023). Applying the same to NSAIDs, the given work intends to explore the receptor-based mechanisms that are associated with the observed cardiovascular risks; thereby, enhancing the understanding of the safety aspects of NSAIDs. Also, classification studies of the SOCs associated with the cardiovascular system were carried out and target-pharmacovigilance signals’ interactions were also analyzed to understand possible receptor mechanisms. In summary, the key strength of our study lies in the integration of pharmacovigilance data with pharmacodynamic insights, following the previously described strategies used in investigating AEs associated with the drugs of the same pharmacological class. This has the advantage of providing a comprehensive exploration of receptor-based mechanisms underlying cardiovascular risks of NSAIDs within different cardiovascular-related SOCs, which helps to add to the knowledge base of the safety of these products.

However, it is essential to understand the limitations of the present research. Firstly, the nature of the database used for self-reporting, namely FAERS, may lead to the problem of imprecision in the analysis. For instance, there is a possibility of missing data in the early paper-based records within FAERS. Besides, the spontaneous reporting system is susceptible to various factors that can result in underreporting, omission, and overall underestimation (Hazell and Shakir, 2006). Due to the unavailability of the reports, sometimes data can be imprecise in terms of weight and onset time, which could distort the results. Secondly, it is always a limitation in all the pharmacovigilance and other observational cohort studies not to establish a causal relationship between reported AEs and drug exposure (Liu et al., 2024). Despite these limitations, disproportionality analysis remains a valuable method for identifying rare signals and monitoring drug safety. Most of the signals that have initially indicated the unsafety of certain drugs have stemmed from disproportionality observed in FAERS. Furthermore, the exact mechanisms of action of drugs that interact with specific receptors and the relation of such interactions to cardiovascular incidents are still partly unknown. The lack of sufficient samples and data on the occupancy of other receptors represents a limitation in the current study. The utilization of multiple NSAIDs was linked to a heightened risk of reporting cardiovascular events, indicating a multifactorial mechanism and synergistic effects that necessitate additional investigation.

## 5 Conclusion

Through the use of pharmacovigilance and pharmacodynamics, this study underlines the significance of continuous monitoring of the cardiovascular risk associated with NSAIDs. In conclusion, our findings, particularly the results of disproportionality analysis, offer significant information on the cardiovascular safety of these generally used NSAIDs. The direct correlation between receptor occupancy levels and the risk of cardiovascular adverse events underscores the significance of considering pharmacodynamic properties in drug safety assessment. The selectivity of NSAIDs for COX-2 and COX-1 can have a significant impact on their cardiovascular safety. Higher COX-2 receptor occupancy is associated with an increased cardiovascular risk from NSAIDs, while higher COX-1 receptor occupancy is linked to a reduced cardiovascular risk from NSAIDs. By integrating pharmacovigilance and pharmacodynamic analyses in our study, we were able not only to relate the detailed receptor mechanisms of NSAIDs on COX isozymes with broader safety signals of SOCs. This detailed consideration provides further clarity on the safety of NSAIDs stressing how maintaining an appropriate balance between COX-1 and COX-2 inhibition reduces cardiovascular events. Continuous post-marketing surveillance and further long-term investigations are imperative to gain a deeper understanding of the potential risks associated with NSAIDs, particularly given their widespread use for pain and inflammation management.

## Data Availability

The original contributions presented in the study are included in the article/Supplementary Material, further inquiries can be directed to the corresponding authors.
