# Supplementary material for: Insights from pharmacovigilance and pharmacodynamics on cardiovascular safety signals of NSAIDs
Source: Front Pharmacol. 2024 Sep 4;15:1455212. doi: 10.3389/fphar.2024.1455212 (PMC11408209; doi:10.3389/fphar.2024.1455212)
Supplement: Supplementary file 2 [file Table2.DOCX]

**Table S1. The UniProt protein ID numbers and their corresponding meanings.**

| UniProt ID | Target Name |
| --- | --- |
| P00915 | Carbonic anhydrase 1 |
| P00918 | Carbonic anhydrase 2 |
| P23219 | Prostaglandin G/H synthase 1 |
| P35354 | Prostaglandin G/H synthase 2 |
| O43570 | Carbonic anhydrase 12 |
| Q8N1Q1 | Carbonic anhydrase 13 |
| Q9ULX7 | Carbonic anhydrase 14 |
| P07451 | Carbonic anhydrase 3 |
| P22748 | Carbonic anhydrase 4 |
| P35218 | Carbonic anhydrase 5A, mitochondrial |
| Q9Y2D0 | Carbonic anhydrase 5B, mitochondrial |
| P23280 | Carbonic anhydrase 6 |
| P43166 | Carbonic anhydrase 7 |
| Q16790 | Carbonic anhydrase 9 |
| P02768 | Albumin |
| P08684 | Cytochrome P450 3A4 |
| P00374 | Dihydrofolate reductase |
| P12104 | Fatty acid-binding protein, intestinal |
| P19224 | UDP-glucuronosyltransferase 1-6 |
| P22309 | UDP-glucuronosyltransferase 1A1 |
| Q9HAW8 | UDP-glucuronosyltransferase 1A10 |
| Q9HAW7 | UDP-glucuronosyltransferase 1A7 |
| O60656 | UDP-glucuronosyltransferase 1A9 |
| P43116 | Prostaglandin E2 receptor EP2 subtype |
| Q9Y6L6 | Solute carrier organic anion transporter family member 1B1 |
| P11712 | Cytochrome P450 2C9 |
| Q8TCC7 | Solute carrier family 22 member 8 |
| Q04828 | Aldo-keto reductase family 1 member C1 |
| P52895 | Aldo-keto reductase family 1 member C2 |
| P42330 | Aldo-keto reductase family 1 member C3 |
| P21554 | Cannabinoid receptor 1 |
| P34972 | Cannabinoid receptor 2 |
| Q04760 | Lactoylglutathione lyase |
| Q9Y5Y4 | Prostaglandin D2 receptor 2 |
| P27169 | Serum paraoxonase/arylesterase 1 |
| Q9NPD5 | Solute carrier organic anion transporter family member 1B3 |
| O94956 | Solute carrier organic anion transporter family member 2B1 |
| P52209 | 6-phosphogluconate dehydrogenase, decarboxylating |
| P11413 | Glucose-6-phosphate 1-dehydrogenase |
| P00390 | Glutathione reductase, mitochondrial |
| Q9NSA0 | Solute carrier family 22 member 11 |
| Q4U2R8 | Solute carrier family 22 member 6 |
